# Effect of the High-Pressure Hydrogen Gas Exposure in the Silica-Filled EPDM Sealing Composites with Different Silica Content

**DOI:** 10.3390/polym14061151

**Published:** 2022-03-13

**Authors:** Hyun Min Kang, Myung Chan Choi, Jin Hyok Lee, Yu Mi Yun, Jin Sub Jang, Nak Kwan Chung, Sang Koo Jeon, Jae Kap Jung, Ji Hun Lee, Jin Hong Lee, Young Wook Chang, Jong Woo Bae

**Affiliations:** 1 Rubber Research Division, Department of Elastic Material Research, Korea Institute of Footwear & Leather Technology, Busan 47154, Korea; khmin0402@naver.com (H.M.K.); mcchoi@kiflt.re.kr (M.C.C.); jhlee@kiflt.re.kr (J.H.L.); ymyun@kiflt.re.kr (Y.M.Y.); 2 School of Chemical Engineering, Pusan National University, Busan 46241, Korea; jinhong.lee@pusan.ac.kr; 3 Department of Materials & Chemical Engineering, Hanyang University, Ansan 15588, Korea; ywchang@hanyang.ac.kr; 4 Research Team of Material Compatibility to Hydrogen Facility, Korea Research Institute of Standards and Science, Daejeon 34113, Korea; jjs77715@kriss.re.kr (J.S.J.); nk.chung@kriss.re.kr (N.K.C.); 5 Department of Materials Science & Engineering, Chungnam National University, Daejeon 34134, Korea; 6 Hydrogen Energy Materials Research Center, Korea Research Institute of Standards and Science, Daejeon 34113, Korea; sangku39@kriss.re.kr (S.K.J.); jkjung@kriss.re.kr (J.K.J.); ljh93@kriss.re.kr (J.H.L.)

**Keywords:** EPDM, silica filler, physical and mechanical properties, resistance to high-pressure hydrogen gas

## Abstract

With the increasing interest in hydrogen energy, the stability of hydrogen storage facilities and components is emphasized. In this study, we analyzed the effect of high-pressure hydrogen gas treatment in silica-filled EPDM composites with different silica contents. In detail, cure characteristics, crosslink density, mechanical properties, and hydrogen permeation properties were investigated. Results showed that material volume, remaining hydrogen content, and mechanical properties were changed after 96.3 MPa hydrogen gas exposure. With an increase in the silica content, the crosslink density and mechanical properties increased, but hydrogen permeability was decreased. After treatment, high-silica-content composites showed lower volume change than low-silica-content composites. The crack damage due to the decompression caused a decrease in mechanical properties, but high silica content can inhibit the reduction in mechanical properties. In particular, EPDM/silica composites with a silica content of above 60 phr exhibited excellent resistance to hydrogen gas, as no change in their physical and mechanical properties was observed.

## 1. Introduction

Renewable and sustainable energies based on solar, wind, bio-based fuel, geothermal power, and hydrogen have attracted attention due to the exhaustion of nonrenewable fossil fuels and global warming caused by greenhouse gas (GHG) emissions. Hydrogen energy is considered a strong candidate for next-generation energy systems because it can be manufactured from plentiful water molecules and produce only water during consumption in a fuel cell. For practical application of hydrogen energy, hydrogen gas is compressed to high pressure due to its very low volume energy density. In fuel cell vehicles (FCVs) and hydrogen fuel stations (HFSs), the technology of hydrogen storage and transportation under 70–90 MPa hydrogen gas is important [1,2,3,4,5].

FCVs and HFSs are composed of various devices, such as hydrogen tanks, valves, pipes, compressors, accumulators, filters, and nozzles. The connection of these devices requires the use of high-pressure hydrogen sealing. Rubber materials are used as seals for O-rings, gaskets, and packing for high-pressure hydrogen gas due to their low hydrogen permeation properties and excellent thermal, chemical resistance, and elasticity [6,7,8]. The representative rubber materials used as sealing devices for FCVs and HFSs are fluoroelastomer (FKM), acrylonitrile butadiene rubber (NBR), and ethylene propylene diene monomer (EPDM). Because each FCV and HFS device is exposed to different temperatures and pressures of hydrogen gas, suitable rubber materials for the various conditions of hydrogen exposure are needed. For example, the sealing devices made of FKM rubber are used for devices of FCVs and HFSs such as hydrogen compressors, which require an excellent resistance for hydrogen gas with high temperature and pressure. Because FKM exhibits excellent thermal and chemical resistance and gas barrier properties and the service temperature range of FKM is from −40 to 225 °C, which is much higher than that of other rubber materials [8], a few studies have investigated the effect of high-pressure hydrogen gas on the physical and mechanical properties of FKM composites and the long-term durability of FKM O-rings [9,10]. Furthermore, NBR is also widely used for seals and fuel hoses in FCVs and HFSs due to its excellent thermal and oil resistance. Several studies have reported the effect of the pressure and temperature of hydrogen gas on the chemical, physical, and mechanical properties of NBR composites filled with carbon black and silica [2,11,12,13].

EPDM has excellent heat and chemical resistance, low-temperature properties, flexibility over a wide temperature range (−40 to 150 °C), and good hydrogen gas barrier properties [14,15,16]. Therefore, EPDM is the most widely used material for seals, tubes, and gaskets in FCVs and HFSs.

In general, exposure to high-pressure hydrogen gas causes mechanical damage to the sealing materials made of rubber, including EPDM. The mechanical damage such as internal cracks called blister fractures reduces the durability of sealing material and is affected significantly by hydrogen exposure conditions such as hydrogen pressures, temperatures, cycle patterns. Several studies have reported the damage evolution process of unfilled EPDM in response to high-pressure hydrogen gas. Yamabe et al. [17,18,19,20] and Koga et al. [7,21] investigated the influence of hydrogen pressure and ambient temperature on fracture behavior of unfilled EPDM. They observed that the degree of blister fracture of unfilled EPDM became more serious as the hydrogen pressure and ambient temperature increased. Furthermore, Yamabe et al. [22] reported that the number of exposure cycles consisting of compressing and decompressing hydrogen gas incorporated with decompression time, pressure, and temperature has a significant impact on the internal cracks in unfilled EPDM.

The type and content of filler also cause mechanical damage to the sealing materials after exposure to high-pressure hydrogen gas. Yamabe et al. [16,23] and Nishimura [5] investigated the effects of filler type and content on hydrogen penetration properties and blister fracture behavior after exposure to 10 MPa hydrogen gas. With increasing filler content, the degree of blister fracture of EPDM composites decreased due to the reinforcing effect of filler. Furthermore, comparing EPDM composites filled with silica and carbon black, the silica-filled EPDM composites exhibited a much lower amount of absorbed hydrogen content than the EPDM composites filled with carbon black. In particular, in the EPDM composites filled with 30 and 60 phr of silica, the blister facture by exposure to high-pressure hydrogen gas was not observed. These results indicated that the addition of silica is an effective way to enhance the resistance to high-pressure hydrogen gas of rubber composites.

Nowadays, there is much interest in increasing the charging speed of hydrogen gas to expand the application of hydrogen energy to commercial vehicles used in the transportation industry. Because the Joule–Thomson coefficient of hydrogen gas is lower than 0 K·bar^−1^ below 200 K, charging a hydrogen storage tank with compressed hydrogen gas causes the temperature of the internal tank to increase rapidly and decreases the charging rate of compressed hydrogen gas due to an increase in the mobility of hydrogen gas at the entrance of the storage tank. The temperature of the compressed hydrogen gas used in FCVs and HFSs is −40 °C. To increase the charging speed of hydrogen gas, it is necessary to decrease the temperature of compressed hydrogen gas to lower than −50 °C. In order to improve the stability of the hydrogen system at lower than −50 °C, it is essential to develop a rubber sealing material with excellent elasticity at low temperatures.

In this study, isooctyl tallate was employed as a plasticizer to lower the glass transition temperature of EPDM composites and improve the flexibility and elasticity of the composites below −50 °C. In addition, silica was employed as a reinforcing filler, which decreases the amount of hydrogen gas absorbed by the EPDM composite and enhances the resistance to high-pressure hydrogen gas. The silica-filled EPDM composites with plasticizers were prepared with varying silica contents by using the peroxide cure system. The effects of silica on the crosslink density, mechanical properties, and hydrogen permeation properties of the composites were investigated before exposure to hydrogen gas. After exposure to 96.3 MPa high-pressure hydrogen gas for 24 h, the volume change, remaining hydrogen content, and the change in mechanical properties of the vulcanizates were investigated.

## 2. Materials and Methods

### 2.1. Materials

Ethylene propylene diene rubber (EPDM, NORDEL IP 4760P, ethylene content: 67.5%, ethylidenenorbornene content: 5%, The Dow Chemical Company, Midland, TX, USA) was used as a raw rubber. Precipitated silica (Zeosil 175MP, BET area: 175 m^2^/g, Solvay, Brussels, Belgium) was used as a reinforcing filler, and bis-(γ-triethoxysilylpropyl)tetrasulfide (TESPT, Nanjing Aocheng Chemical Ltd., Jiangsu, China) was used as a silane coupling agent. Polyethylene glycol (PEG-4000, MW: 3180–3520, Green Chemical Ltd., Seosan, Korea) was used as an activator. Isooctyl tallate (Hallstar, Chicago, IL, USA) was used as a plasticizer. Dicumyl peroxide (DCP, Perkadox BC-FF, Nouryon, Amsterdam, Netherlands) was used as the curing agent. Triallyl cyanurate (TAC, FARIDA TACE, Hunan Farida Technology Co., Ltd., Changsha, China) was used as an accelerator. Zinc oxide and stearic acid were supplied by PJ CHEMTEK Ltd., Yangsan, Korea, and LG Household & Health Care, Seoul, Korea, respectively.

### 2.2. Preparation of Silica-Filled EPDM Composites with Different Silica Contents

The EPDM/silica composites were prepared with different silica contents: 0, 20, 40, 60, 80, and 100 phr (parts per hundred parts of rubber). The detailed formulation is listed in Table 1. The peroxide curing system was employed for the preparation of vulcanized EPDM/silica composites. For simplicity, the EPDM/silica composites are named Sx, where x indicates the phr content of the silica filler. For example, S60 is EPDM composites filled with 60 phr of silica.

The composites were prepared by using a two-step mixing process. In the first mixing step, EPDM rubber was mixed with precipitated silica, plasticizer, processing aids, and silane coupling agent in an internal mixer (3 L kneader, Moriyama Co., Tokyo, Japan) with a starting temperature of 80 °C and a mixing dump temperature of 140 °C using a rotor speed of 30 rpm for 15 min. In the second mixing step, curing agent and accelerator were added into the mixture obtained in the first mixing step by using 8-inch two open roll mills (PK-RM20140930, Pungkwang Co., Hwaseong, Korea).

Then, vulcanizate sheets were prepared by compression molding in a hot press at 160 °C. Compression molding time employed t_90_ was determined by moving die rheometer (MDR, JIS K63002-2, Nichigo Shoji Co., Ltd., Tokyo, Japan).

### 2.3. Curing Behavior

Curing behavior and characteristics were analyzed using MDR according to the ASTM D 2084 standard at 160 °C. The oscillation frequency was 100 cycles min^−1^ (1.66 Hz) with an amplitude of 1°.

### 2.4. Crosslink Density

To obtain the crosslink density of the vulcanizates, a volume swelling test was performed. A rectangular-shaped sample with dimensions of 25 × 25 × 2 mm^3^ was put into tetrahydrofuran (THF) for 72 h at room temperature and taken out. After cleaning the adhering THF from the surface of the samples, the swollen samples were weighed immediately. The weights of the samples were measured by using an electronic balance with an accuracy of 0.01 g. The volume swelling ratio (*V_Q_*) was determined from the weight of the sample in the unswollen and swollen states using the following equation:

(1) $$V_{Q}\left(\%\right)=\frac{\left(W_{s}-W_{d}\right)/\rho_{s}}{W_{d}/\rho_{r}}\times 100$$

where *V_Q_* is the volume swelling ratio, *W_d_* is the initial weight of the sample, *W_s_* is the weight of the sample in the swollen state, *ρ_r_* is the density of the EPDM, and *ρ_s_* is the density of the THF.

The crosslink density was calculated according to the Flory–Rehner equation as follows [24,25]:

(2) $$V=\frac{1}{2M_{C}}=-\frac{\ln\left(1-V_{1}\right)+V_{1}+\chi V_{1}^{2}\;}{2\rho_{r}V_{0}\left(V_{1}^{\frac{1}{3}}-\frac{V_{1}}{2}\right)}$$

(3) $$V_{1}=\frac{\frac{W_{d}-W_{f}}{\rho_{r}}}{\frac{W_{d}-W_{f}}{\rho_{r}}+\frac{W_{s}-W_{d}}{\rho_{s}}}$$

where *V* is the crosslink density (mol/g), *M_C_* is the average molecular weight between crosslink points (g/mol), *V*_0_ is the molar volume of solvent (cm³/mol), *V*_1_ is the volume fraction of rubber in the swollen gel at equilibrium, *W_d_* is the weight of the unswollen sample, *W_f_* is the weight of the filler in the sample, *W_s_* is the weight of the swollen sample, *ρ_r_* is the density of EPDM, *ρ_s_* is the density of THF, and *χ* is the polymer–solvent interaction parameter (*χ* = 0.501) [26].

### 2.5. Mechanical Properties

The hardness of the vulcanizates was measured using a Shore A durometer (ASKER Type A, KOBUNSHI KEIKI Co., Ltd., Kyoto, Japan) according to the ASTM D 2240 standard. The mechanical properties of vulcanizates were measured using a universal testing machine (UTM, Instron 3345, Instron Ltd., Norwood, MA, USA) according to test method A in ASTM D 412 at room temperature. The shape and dimension of test specimens were Die C type. The crosshead speed was 500 mm/min, and at least 5 samples were used for measuring the mechanical properties.

### 2.6. Dynamic Mechanical Thermal Analysis

The dynamic mechanical properties were analyzed using a dynamic mechanical analysis (DMA, DMA-Q800, TA instruments, New Castle, DE, USA). The test temperature range was from −100 to 100 °C with a heating rate of 2 °C min^−1^. The sample was subjected to an amplitude of 40 µm at a frequency of 1 Hz. The results are presented in terms of storage modulus and hysteresis (tan δ) according to temperature.

### 2.7. Hydrogen Permeation Properties

The hydrogen permeation properties were measured by using a differential press method based on Fick’s diffusion law and Henry’s gas solubility law. Samples were punched out in a round shape with a diameter of 35 mm from the vulcanizate sheet. The test was carried out with 99.9999% hydrogen gas at ambient temperature. The samples were put in the space between the upper and lower cell, and the porous support was employed to maintain a constant gas-permeable area. Both the upper and lower cells were evacuated to a pressure of 0.1 kPa and 0.001 kPa, respectively. After the hydrogen gas was pumped into the upper cell until it reached a pressure of 100 kPa, the pressure in the lower cell was recorded and graphed until it reached a pressure of 1.3 kPa. Based on the obtained results, the hydrogen permeability coefficient (P), diffusivity coefficient (D), and solubility coefficient (S) of samples were calculated [27].

### 2.8. Remaining Hydrogen Content

The maximum hydrogen exposure of 96.3 MPa was achieved by using 99.999% hydrogen gas in a high-pressure cylindrical chamber for 24 h at ambient temperature. After installing the samples in the chamber, a hydrogen purge was performed at 5 MPa three times to achieve the removal of other gases. After the purge, the chamber was filled with hydrogen gas at a rate of approximately 5 MPa/min to a pressure of up to 96.3 MPa. Hydrogen gas exposure for 24 h is sufficient to attain the equilibrium state for the gas sorption of a sample about 2.5 mm in thickness. Decompression to atmospheric pressure was conducted at a rate of approximately 1 MPa/s. After decompression, the samples were removed from the chamber and loaded into the gas space of a graduated cylinder.

The remaining hydrogen content was determined using the volumetric analysis technique by employing a graduated cylinder and diffusion analysis program, which was developed recently [28,29].

### 2.9. The Volume Change and Mechanical Properties of the Vulcanizate after High-Pressure Hydrogen Gas Exposure

Material volume change was measured both before hydrogen exposure and after exposure to hydrogen gas at pressures of up to 96.3 MPa for 24 h at room temperature. The volume change values (Δ*V*) of the EPDM/silica vulcanizates at 1 h or 24 h after decompression were obtained as follows:

(4) $$\Delta V\;\left(\%\right)=\frac{V_{f}-V_{i}}{V_{i}}\times 100$$

where *V_f_* is the volume of the sample at 1 h or 24 h after decompression and *V_i_* is the initial volume of the sample before exposure to hydrogen gas.

The tensile test was conducted to investigate the effect of exposure to high-pressure hydrogen gas on the mechanical properties of vulcanizates by using standard test specimens in accordance with Die C type of ASTM D 412. To obtain the test specimens for the vulcanizates after exposure to high-pressure hydrogen gas, the compression-molded sheet with dimensions of 150 mm × 150 mm × 2 mm was put into the chamber filled with 96.3 MPa hydrogen gas for 24 h. After decompression, standard test specimens were prepared by cutting from the swollen sheets. The change in the mechanical properties (Δ*M*) of EPDM/silica composites at 1 h or 24 h was obtained as follows:

(5) $$\Delta M\;\left(\%\right)=\frac{M_{A}-M_{B}}{M_{B}}\times 100$$

where *M_A_* is the mechanical properties at 1 h or 24 h after decompression and *M_B_* is the initial mechanical properties before exposure to hydrogen gas.

### 2.10. Image Analysis of the Fractured Surface of Hydrogen Exposure Vulcanizate

The fractured surfaces of the vulcanizate were observed by field-emission scanning electron microscopy (FE-SEM; JSM-6701F, JEOL Ltd., Tokyo, Japan) at an accelerating voltage of 15 kV under an N_2_ atmosphere. The samples derived from mechanical properties testing before hydrogen exposure and 24 h after decompression were used for the observation of fractured surfaces. The fractured surface was sputter-coated with gold.

## 3. Results and Discussion

### 3.1. Curing Characteristics

**Figure 1:**
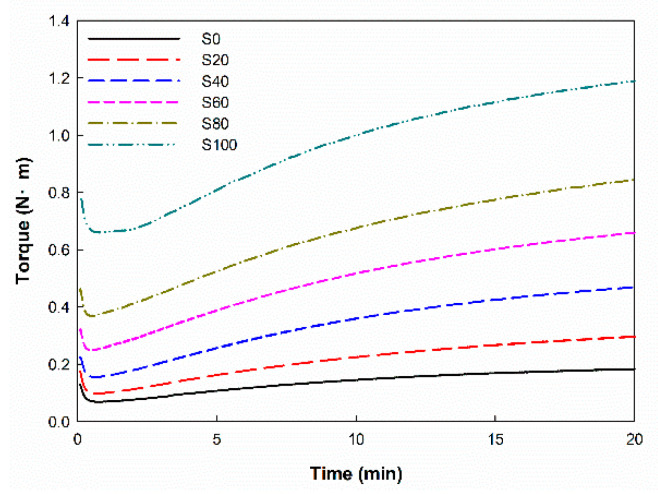
Curing behavior of the EPDM/silica composites.

Figure 1 and Table 2 show the curing characteristics of EPDM/silica composites with different silica contents. The minimum torque (M_L_) and maximum torque (M_H_) of the silica-filled EPDM increased as the silica content increased. The M_L_ was increased because the viscosity increased as silica content increased [30]. The M_H_ increased owing to the increased degree of crosslink. The delta torque (ΔT = M_H_ − M_L_) increases as the silica content increases owing to the reinforcing effect of the silica [30]. The optimum cure time (t_90_) of the silica-filled EPDM increased more than that of unfilled EPDM. This is due to the fact that silica reacts with accelerators and activators during compounding, causing cure retardation [31]. However, it was similar with regard to the silica content. The cure rate of the silica-filled EPDM composites increased as the silica content increased.

### 3.2. Swelling Ratio and Crosslink Density of EPDM/Silica Vulcanizates

**Figure 2:**
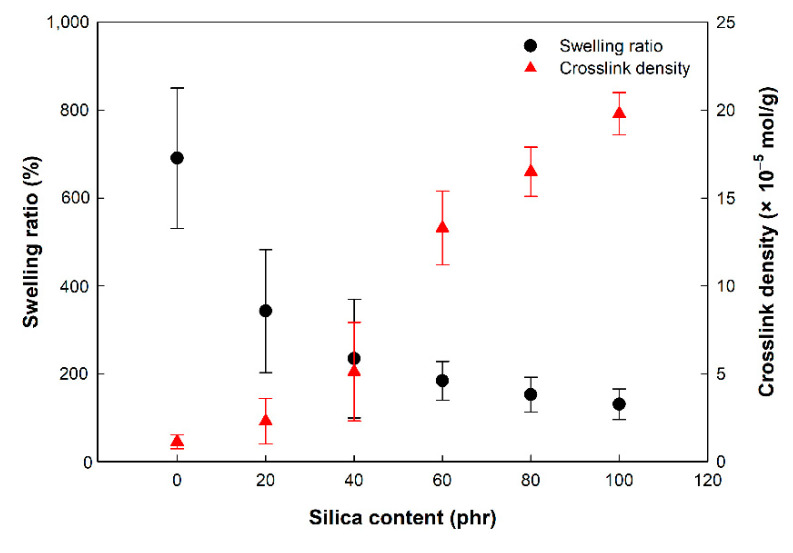
Swelling ratio and crosslink density of EPDM/silica vulcanizates.

The swelling ratio and crosslink density of the EPDM vulcanizates are shown in Figure 2. The swelling ratio of the EPDM/silica composites decreased as the silica content increased. The swelling ratio value of the unfilled EPDM was 690.7%, and that of the vulcanizates with 20~100 phr silica contents was 130.3~342.4%. This tendency occurred due to increased crosslink density.

For quantitative evaluation, the crosslink density was measured using the Flory–Rehner equation. The crosslink density of the EPDM/silica vulcanizates increased as the silica contents increased. An increase in the silica content caused an increase in the filler–rubber interaction (F-R interaction) between the silica and EPDM chain due to the silane coupling agent, which decreases the hydrophilic character of the silica surface [32]. These crosslinks reduce the extensibility of the rubber chains caused by swelling and make it more difficult for a solvent to permeate between rubber molecules since the crosslink density is inversely proportional to the swelling ratio [33].

### 3.3. Physical and Mechanical Properties of EPDM/Silica Vulcanizates

**Figure 3:**
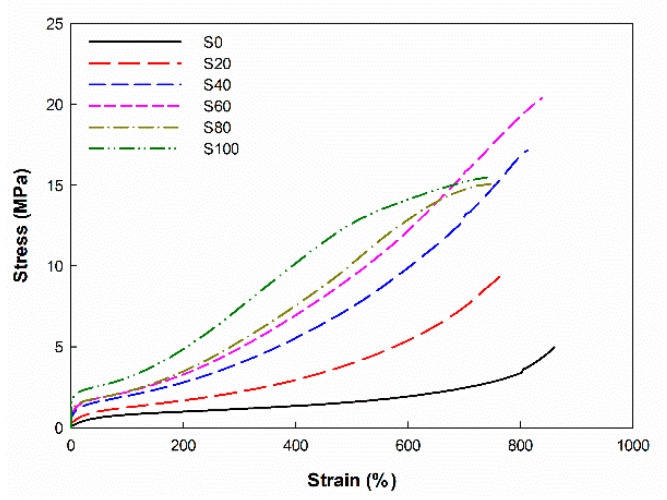
Stress–strain curve of the EPDM/silica vulcanizates.

The stress–strain curve and the values for the hardness, tensile strength, and modulus of silica-filled EPDM vulcanizates are shown in Figure 3 and Table 3. Clearly, the hardness, tensile strength, and modulus of the silica-filled vulcanizates increased as the silica content increased. When silica was added, the tensile strength of the composite became about 3 to 6 times higher than that of the unfilled EPDM composite owing to the F-R interaction. The increase in the tensile strength caused by increasing the silica content occurred owing to the increased reinforcement and increased crosslink density [30]. The tensile strength of silica-filled EPDM increased with the silica content until a maximum level was reached at 60 phr of silica content; then, this property started to decrease as the silica content increased due to the dilution effect or agglomeration of silica filler. In addition, EPDM would not sufficiently retain silica due to the increase in silica volume fraction with increasing silica content. The mechanical behavior of other filler vulcanizates followed a similar pattern [34,35].

The elongation at the break decreased as the silica content increased. In general, because crosslink density is inversely proportional to elongation at the break, vulcanizates with higher crosslink density have shorter elongation at the break than those with a lower crosslink density [30].

The 100% modulus increased as the silica content increased, as shown in Table 3. The crosslink density was related to the modulus of the rubber vulcanizates. EPDM/silica vulcanizates with a higher crosslink density had a larger modulus than EPDM/silica vulcanizates with a lower crosslink density [30].

### 3.4. Dynamic Mechanical Analysis of EPDM/Silica Vulcanizates

**Figure 4:**
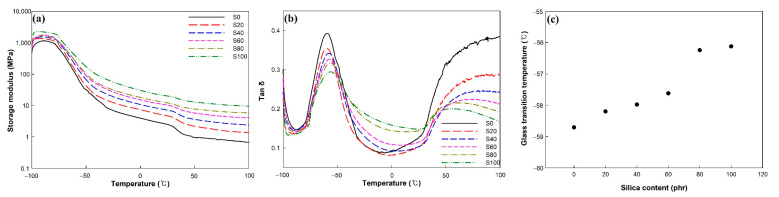
Dynamic mechanical analysis of the EPDM/silica vulcanizates: (**a**) storage modulus, (**b**) tan δ, and (**c**) glass transition temperature.

The DMA results for the EPDM/silica vulcanizates are shown in Figure 4a,b. Figure 4a shows the storage modulus with temperature for the silica-filled vulcanizates. Over the whole temperature range, the storage modulus of EPDM/silica vulcanizates was higher than that of the unfilled EPDM vulcanizates. For example, the storage modulus of S100 at −40 °C and 85 °C was 6.3 and 13.3 times higher than that of the unfilled EPDM, respectively. The silica effectively strengthened the rubber matrix, according to these results.

Figure 4b,c show the tan δ and glass transition temperature (T_g_) of the EPDM/silica vulcanizates. The peak tan δ at T_g_ decreased due to decreased rubber fraction with increasing silica content [36]. The tan δ in the temperature range of −20 to 50 °C increased due to crystallite melting [37]. The melting of crystalline decreased storage modulus and increased loss modulus. The T_g_ of the EPDM/silica vulcanizates slightly increased as the silica content increased because of the increase in the crosslink density, which caused the mobility of the polymer chain to decrease. The T_g_ of the EPDM/silica vulcanizates was −58.7 to −56.1 °C. These values are about 25~28 °C lower than those of a general EPDM composite with a similar chemical composition (ethylene/propylene ratio of 69/30.5 wt%) since we used plasticizer, which facilitates the incorporation of fillers, improves low-temperature flexibility, and provides a softer vulcanizate [14,38].

The EPDM/silica vulcanizates had an excellent elasticity below −50 °C. Therefore, they could be used as sealing materials that are required to have a hydrogen sealing performance.

### 3.5. Hydrogen Permeation Properties of EPDM/Silica Vulcanizates

**Figure 5:**
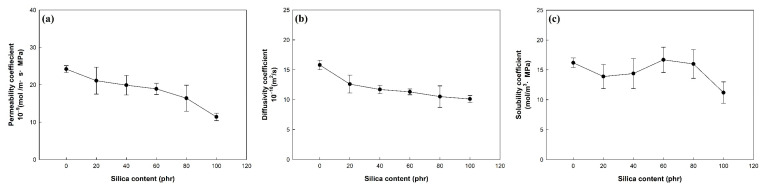
Hydrogen permeation properties of EPDM vulcanizates under 1 atm of hydrogen gas: (**a**) permeability coefficient, (**b**) diffusivity coefficient, and (**c**) solubility coefficient.

Figure 5 and Table 4 show the permeability, diffusivity, and solubility coefficient for the hydrogen gas of the EPDM/silica vulcanizates. As shown in Figure 5a, the permeability coefficient of silica-filled EPDM vulcanizates is lower than that of unfilled EPDM vulcanizates. In silica-filled EPDM vulcanizates, the permeability coefficient decreased with increasing silica contents, because the silica reduced the weight fraction of rubber that hydrogen can impregnate [2]. Furthermore, the permeability coefficient was related to the crosslink density. As the crosslink density increased, the mobility of the rubber chains decreased and the values of the hydrogen gas barrier properties increased. The permeability coefficient decreased in vulcanizates that had a high crosslink density [39].

Figure 5b shows the diffusivity coefficient of the EPDM/silica vulcanizates. The diffusivity coefficient of the EPDM/silica vulcanizates decreased as the silica content increased, which may have occurred because of the prolonged diffusion path caused by the addition of silica [28]. Figure 5c shows the solubility coefficients of the filled EPDM. The solubility coefficients of the EPDM/silica vulcanizates were within the range of 11 to 17 mol/m^3^·MPa regardless of the silica content.

### 3.6. Volume Change and Remaining Hydrogen Content of EPDM/Silica Vulcanizates after High-Pressure Hydrogen Gas Exposure

**Figure 6:**
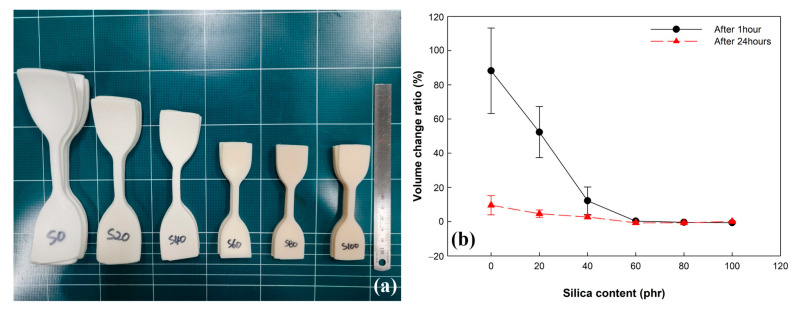
Effects of silica content on the volume changes of EPDM after exposure to 96.3 MPa of hydrogen gas for 24 h: (**a**) the image immediately after decompression and (**b**) volume changes after decompression.

The swelling behavior caused by high-pressure hydrogen gas in rubber composites was a significantly concerning factor for designing the sealing parts of HFCVs and HFSs. The volume change of the EPDM/silica vulcanizates at 1 h and 24 h after decompression was shown in Figure 6. As shown in Figure 6a, it visually confirms that the volume of vulcanizates with a lower silica content than 60 phr increases after exposure to high-pressure hydrogen gas, and the volume change is very dependent on the amount of silica filler in the vulcanizates.

At 1 h after decompression, the volume change of unfilled EPDM (S0), S20, and S40 is 88%, 52%, and 12%. Above 60 phr of silica content, the volume change of vulcanizates is lower than 1%, which makes it difficult to compare the volume change according to silica content. The decrease in the volume change of vulcanizates with increasing the silica content results from the reinforcing effect of silica fillers, which suppress the volume expansion. The results are consistent with previously reported studies [5].

At 24 h after decompression, the volume change of vulcanizates filled with lower silica content than 40 phr is lower than that at 1 h after decompression. In particular, S0 and S20 are 9.6% and 4.6%, respectively, which are much lower than that at 1 h after decompression. Above 60 phr of silica content, the volume change of vulcanizates is similar to that at 1 h after decompression because that is not affected by exposure to high-pressure hydrogen. The volume change of vulcanizates decreased with increasing the time after decompression due to the release of the dissolved hydrogen molecules in vulcanizates driven by the difference of pressure.

**Figure 7:**
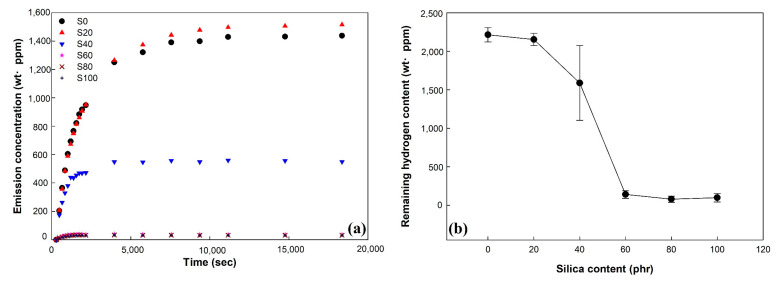
Emission concentration and remaining hydrogen content of EPDM/silica vulcanizates exposed to 96.3 MPa pressure hydrogen gas: (**a**) emission concentration and (**b**) remaining hydrogen content.

To identify the volume change of the vulcanizates in more detail, the remaining hydrogen content in the vulcanizates was measured and is shown in Figure 7.

As shown in Figure 7a, the hydrogen emission concentration of vulcanizates increased with increasing time after decompression. After 10,000 s, the hydrogen emission concentration of vulcanizates reached its maximum value, which was constant regardless of time. With increasing silica content, the maximum value and the time for reaching of hydrogen emission concentration of vulcanizates decreased. This indicated that the hydrogen emission concentration of vulcanizates is dependent on the content of silica fillers in the vulcanizates. Figure 7b shows the remaining hydrogen content of vulcanizates calculated by using the hydrogen emission concentration. The remaining hydrogen content of vulcanizates decreased as the silica content increased. The remaining hydrogen content values of S0, S20, and S40 were 2215, 2154, and 1589 wt·ppm, respectively. These were relatively large values compared to the remaining hydrogen contents of samples S60, S80, and S100, which were 141, 98, and 79 wt·ppm, respectively. These trends are similar to the results of volume change at 1 h after decompression. It is confirmed that the volume change of vulcanizates is significantly affected by silica loading and remaining hydrogen content.

### 3.7. The Change in Mechanical Properties after High-Pressure Hydrogen Gas Exposure

**Figure 8:**
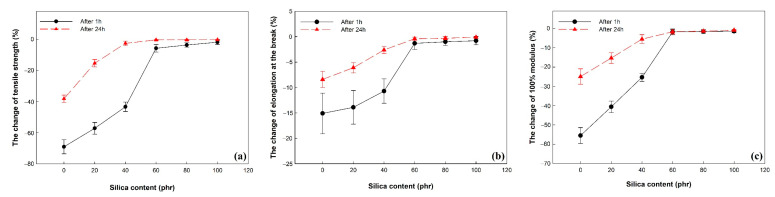
The change in the mechanical properties of EPDM/silica vulcanizates after exposure to 96.3 MPa hydrogen gas for 24 h: (**a**) the change of tensile strength, (**b**) the change of elongation at the break, and (**c**) the change of 100% modulus.

The effect of swelling behavior and remaining hydrogen content on the mechanical properties of vulcanizates was investigated. The change of mechanical properties after exposure to high-pressure hydrogen gas was as shown in Figure 8 and Table 5.

At 1 h after decompression, the change of tensile properties of vulcanizates decreased as the silica content increased. Below the silica loading 60 phr, the decrease in tensile strength and 100% modulus of vulcanizates was larger than the decrease in elongation at the break of vulcanizates. This indicated that dissolved hydrogen molecules acted as a plasticizer for vulcanizates.

At 24 h after decompression, the change of tensile properties of vulcanizates is much lower than that at 1 h after decompression. This indicated that the amount of dissolved hydrogen gas that acted as a plasticizer in vulcanizates decreased with increasing the time after decompression. Above the silica loading 60 phr, the change of tensile properties of vulcanizates was lower than 2%. This indicated that the tensile properties of vulcanizates recovered before exposure to high-pressure hydrogen gas, which is similar to the value of volume change at 24 h after decompression. However, the decrease in tensile properties of vulcanizate filled with lower silica loading than 60 phr is relatively high compared to the volume change of those at 24 h after decompression. During the release of dissolved hydrogen gas, the volume of vulcanizates recovered before exposure to hydrogen gas, but the mechanical properties decreased due to the microbubbles and cracks generated by the cluster of saturated hydrogen gas [16,23].

### 3.8. The Fracture Surfaces of EPDM/Silica Vulcanizates

**Figure 9:**
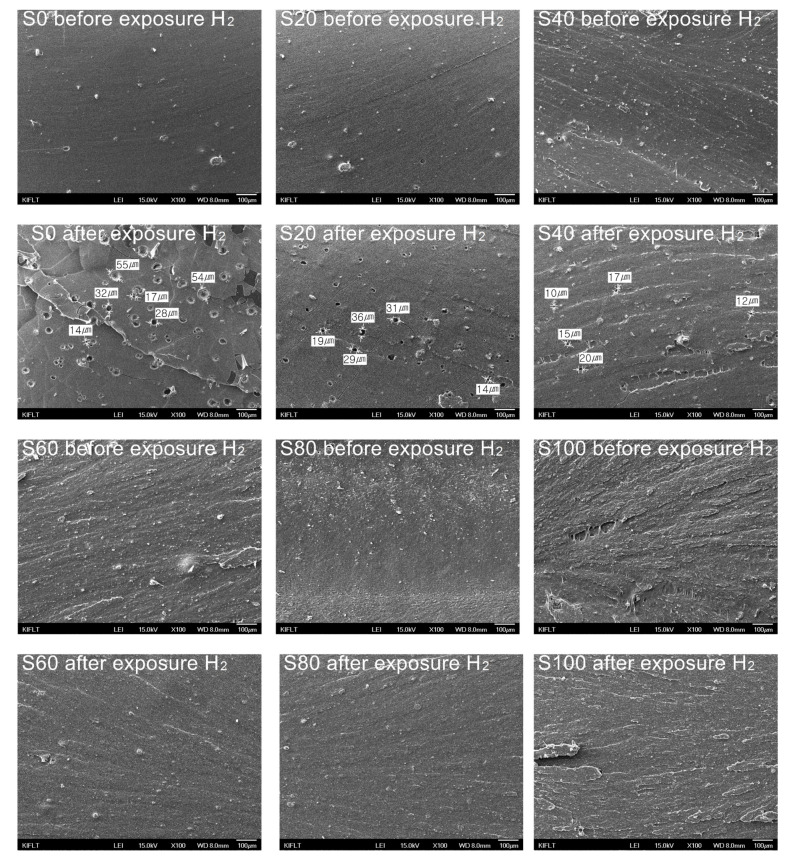
SEM images of the fracture surfaces of EPDM/silica vulcanizates before and after exposure to 96.3 MPa of hydrogen gas (H_2_).

To clarify the decrease in mechanical properties of the vulcanizates after exposure to high-pressure hydrogen gas, the fracture surfaces of the vulcanizates were analyzed by FE-SEM, and the images are shown in Figure 9.

Before exposure to hydrogen gas, all samples did not exhibit any defect in the fracture surface. However, after exposure to hydrogen gas, defects such as voids and cracks were observed on the fracture surface of the vulcanizates filled with silica content than 60 phr. The average sizes of numerous voids on the fracture surface in S0, S20, and S40 were 37.2, 25.8, and 14.8 µm, respectively. The average size and number of voids decreased with increasing silica content. Cracks were also observed around the voids, which is due to the stress concentration by the voids. It is confirmed that the decrease in mechanical properties of vulcanizates was caused by the defects such as voids and cracks which were generated by the release of the dissolved hydrogen gas.

## 4. Conclusions

The silica-filled EPDM composites with plasticizer were manufactured with different silica contents of 0, 20, 40, 60, 80, and 100 phr of EPDM rubber. The crosslink density and the reinforcing effect of the EPDM/silica composites increased as the silica content increased. The addition of silica led to an effective enhancement of the mechanical properties and hydrogen gas barrier properties.

The glass transition temperatures of the vulcanizates were −56 to −58 °C, lower than those of general EPDM. The permeability and diffusivity coefficients of the vulcanizates to hydrogen gas decreased as the silica content increased due to the increase in the crosslink density and prolonged diffusion path caused by the addition of silica. After exposure to high-pressure hydrogen gas, the volume change, remaining hydrogen content, and change in the tensile properties of the vulcanizates decreased as the silica content increased. Above a silica content of 60 phr, the physical and mechanical properties of the vulcanizates were not affected by exposure to high-pressure hydrogen gas.

Therefore, EPDM/silica vulcanizates with high silica content are suitable for use as sealing materials for hydrogen fuel cell vehicles and hydrogen stations, as they have a low glass transition temperature and an excellent resistance to high-pressure hydrogen.

## Figures and Tables

**Table 1:** Formulation of EPDM/silica composites with different silica contents.

Ingredients (phr)	S0	S20	S40	S60	S80	S100
EPDM	100	100	100	100	100	100
Silica	0	20	40	60	80	100
Si-69	0	1.6	3.2	4.8	6.4	8.0
PEG 4000	0	0.8	1.6	2.4	3.2	4.0
Plasticizer	40	40	40	40	40	40
ZnO	3	3	3	3	3	3
Stearic acid	1	1	1	1	1	1
DCP	2.4	2.4	2.4	2.4	2.4	2.4
TAC	1.6	1.6	1.6	1.6	1.6	1.6

**Table 2:** Cure characteristics of the EPDM/silica composites.

Cure Characteristics	S0	S20	S40	S60	S80	S100
M_L_ (N·m)	0.068	0.097	0.154	0.248	0.368	0.625
M_H_ (N·m)	0.184	0.297	0.470	0.660	0.844	1.189
ΔT (N·m)	0.116	0.200	0.316	0.412	0.476	0.564
T_90_ (min)	15.79	16.46	16.28	16.26	16.42	16.03
Cure rate (N·m/min)	0.003	0.012	0.020	0.026	0.030	0.040

**Table 3:** Physical and mechanical properties of the EPDM/silica vulcanizates.

Properties	S0	S20	S40	S60	S80	S100
Hardness (Shore A)	44 ± 2	53 ± 1	60 ± 2	67 ± 2	73 ± 3	80 ± 2
Tensile strength (MPa)	3.6 ± 0.2	10.7 ± 1.0	17.3 ± 0.8	18.9 ± 2.0	16.1 ± 1.0	13.5 ± 0.8
Elongation at break (%)	823 ± 20	811 ± 20	804 ± 25	780 ± 21	753 ± 35	783 ± 20
100% modulus (MPa)	0.9 ± 0.04	1.2 ± 0.01	1.7 ± 0.03	2.0 ± 0.2	2.2 ± 0.1	2.5 ± 0.2

**Table 4:** Summary of the hydrogen gas permeation parameters of the EPDM/silica vulcanizates.

Silica Content (phr)	Permeability Coefficient 10^−9^ (mol/m·s·MPa)	Diffusivity Coefficient 10^−10^ (m^2^/s)	Solubility Coefficient (mol/m^3^·MPa)
0	24.1 ± 0.9	15.8 ± 0.8	16.2 ± 0.8
20	21.1 ± 3.6	12.6 ± 1.5	13.9 ± 2.0
40	19.9 ± 2.6	11.7 ± 0.7	14.4 ± 2.5
60	18.9 ± 1.5	11.3 ± 0.5	16.7 ± 2.1
80	16.4 ± 3.5	10.5 ± 1.8	16.0 ± 2.4
100	11.4 ± 1.0	10.1 ± 0.6	11.2 ± 1.8

**Table 5:** The change in the mechanical properties after exposure to high-pressure hydrogen gas.

Silica Content (phr)	The Change of Tensile Strength (%)		The Change of Elongation at the Break (%)		The Change of 100% Modulus (%)	
	After 1 h	After 24 h	After 1 h	After 24 h	After 1 h	After 24 h
0	−69.0 ± 5.4	−42.3 ± 3.2	−15.1 ± 4.8	−9.2 ± 2.6	−55.5 ± 5.2	−24.9 ± 5.4
20	−57.1 ± 4.1	−27.3 ± 2.8	−13.5 ± 3.1	−7.4 ± 1.9	−30.7 ± 4.6	−13.3 ± 4.8
40	−49.5 ± 3.6	−8.4 ± 1.6	−10.4 ± 2.9	−3.1 ± 0.5	−20.6 ± 2.8	−4.7 ± 2.7
60	−4.6 ± 1.8	−0.4 ± 0.3	−1.7 ± 1.6	−0.4 ± 0.2	−1.8 ± 1.2	−1.8 ± 1.6
80	−3.2 ± 1.6	−0.3 ± 0.2	−1.3 ± 0.9	−0.4 ± 0.3	−1.6 ± 1.1	−1.2 ± 0.9
100	−1.6 ± 1.4	−0.2 ± 0.3	−0.9 ± 0.6	−0.1 ± 0.1	−1.2 ± 0.7	−0.9 ± 0.8

## Data Availability

Not applicable.
